# Large-Sized GaN Crystal Growth Analysis in an Ammonothermal System Based on a Well-Developed Numerical Model

**DOI:** 10.3390/ma15124137

**Published:** 2022-06-10

**Authors:** Pengfei Han, Bing Gao, Botao Song, Yue Yu, Xia Tang, Botao Liu

**Affiliations:** The Institute of Technological Sciences, Wuhan University, Wuhan 430072, China; 2020106520028@whu.edu.cn (P.H.); 2020106520027@whu.edu.cn (B.S.); 2019106520025@whu.edu.cn (Y.Y.); 2018106520022@whu.edu.cn (X.T.); looper2011@163.com (B.L.)

**Keywords:** gallium nitride, ammonothermal system, numerical model, heat source, BP neural net, NSGAII, large-sized GaN growth

## Abstract

The ammonothermal method is considered the most promising method of fabricating bulk gallium nitride (GaN) crystals. This paper improves the ammonothermal growth model by replacing the heater-long fixed temperature boundary with two resistance heaters and considering the real thermal boundary outside the shell. The relationship between power values and temperatures of dissolution and crystallization is expressed by the backpropagation (BP) neural network, and the optimal power values for specific systems are found using the non-dominated sorting genetic algorithm (NSGAII). Simulation results show that there are several discrepancies between updated and simplified models. It is necessary to build an ammonothermal system model with resistance heaters as a heat source. Then large-sized GaN crystal growth is analyzed based on the well-developed numerical model. According to the simulation results, both the increasing rate and maximum stable values of the metastable GaN concentration gradient are reduced for a larger-sized system, which is caused by the inhomogeneity of heat transfer in the autoclave.

## 1. Introduction

With the development of semiconductor technology, gallium nitride (GaN) is becoming increasingly popular due to its advantage of a wide bandgap, which can reach as high as 3.4 eV. Thus, it is widely used in many new fields, such as wireless charging, new-energy vehicles, and optical masers [1,2]. Three main methods are used to grow GaN substrates: the hydride vapor phase epitaxy (HVPE) method [3], the sodium flux method [4], and the ammonothermal method [5]. Among them, the HVPE method is the mainstream method for GaN wafer fabrication. However, its low crystal quality and high cost restrict its application in bulk GaN growth. In contrast, the ammonothermal method can provide high-quality GaN crystals at a low cost and is suitable for volume production [6].

Several groups have conducted both experimental and numerical studies on the ammonothermal growth of bulk GaN. Zajac et al. [7] achieved a 2-inch GaN wafer with a large curvature radius and a low TDD order in an ammonothermal autoclave. Tuomisto et al. [8] studied the defects in bulk GaN grown using the ammonothermal method and proposed several methods to change the defect structure. Kazuo Yoshida et al. [9] developed a high-temperature autoclave using the acidic ammonothermal method to grow GaN crystals. The XRC-FWHM results showed that the yielded crystals were of high quality. Chen et al. [10,11,12] numerically studied the baffle and height of heaters that affect the flow and heat transfer in an ammonothermal autoclave. Hooman Enayati et al. [13,14,15,16] conducted a series of numerical simulations to study the flow and heat transfer in a laterally heated cylindrical reactor, which is similar to an ammonothermal autoclave. Masuda et al. [17] calculated the flow and thermal field in the ammonothermal autoclave with a funnel-shaped baffle. A chemical dissolution reaction in the porous media and on the surface of seed crystals was first introduced by Iman Mirzaee et al. [18], who also used the piecewise linear interface calculation (PLIC) concept to simulate the crystal growth process. Schimmel et al. [19] studied the influence of flow and heat transfer on the temperature measurement error based on experiments. Moreover, several companies from Japan, the United States, and Poland have also greatly contributed to the development of GaN growth in an acidic or basic ammonothermal system, including Ammono (basic system), Mitsubishi Chemical Corp. (acidic system), Soraa Inc. (acidic system), SixPoint Materials Inc. (basic system), and Asahi Kasei Corp. (acidic system) [9,20,21,22].

The heaters of most numerical studies by previous researchers are simplified as heater-long fixed temperatures due to the difficulty of finding corresponding heater powers at certain growth temperatures, which involves a problem of multi-objective optimization. However, according to the recent research by Saskia Schimmel et al. [23], the thermal field using heater-long fixed temperatures as heat sources has a significant deviation from the real temperature distribution in the chamber of the ammonothermal system. In this study, we employ resistance heaters as the heat sources in the ammonothermal numerical model. The relationship between heater powers and the temperatures of dissolution and crystallization is first expressed using a backpropagation (BP) neural net algorithm. Then, the optimal power groups of the two resistance heaters are found using a non-dominated sorting genetic algorithm (NSGAII). A multi-physics field numerical model is established considering mass transport, which is neglected by Saskia Schimmel et al. [23]. Based on the well-developed model, the growth of 2- and 4-inch GaN crystals is analyzed.

## 2. Numerical Model and Mathematical Method

### 2.1. Geometric Model of the Ammonothermal System

**Figure 1:**
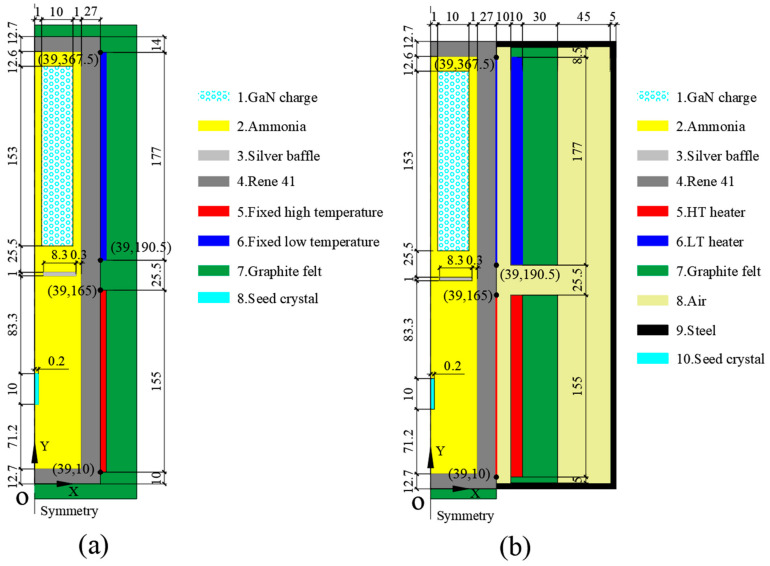
Schematic diagram of the ammonothermal system (unit: mm). (**a**) Original simplified model. (**b**) Updated model using resistance heaters as heat sources.

The model in this study is constructed based on the ammonothermal system used by the Air Force Research Laboratory (Tem-Press MRA 378R). The structure and geometric size of the autoclave are shown in Figure 1. According to our previous research [24], a nutrient basket with a central hole is beneficial for the improvement of the growth rate. Thus, the optimized model is used in this paper. The diameters of the nutrient basket and central hole are 22 mm and 2 mm. The porosity of the nutrient basket is 0.6. A mineralizer of KNH_2_ is adopted, which results in the retrograde solubility of GaN in supercritical ammonia. Thus, the nutrient basket is placed in the upper half of the chamber, 12.6 mm away from the top of the autoclave. The inner height and diameter of the chamber are 356.6 mm and 24 mm, respectively. A circular silver baffle with an 80% opening and seed crystals are located in the middle and lower half of the chamber, respectively. The thickness and diameter of the baffle are 1 mm and 23.4 mm. There is a 1 mm gap between the baffle and the internal face. The distance between the baffle and the nutrient basket’s bottom is 25.5 mm. A seed crystal with 10 mm height and 0.2 mm radius is placed in the middle of the lower half, 83.3 mm away from the baffle. For the original model used by previous researchers, heater-long fixed temperatures are set as the thermal boundary (shown in Figure 1a). High- and low-temperature resistance heaters (HT heater and LT heater) are used as heat sources in our updated model and are placed in the lower and upper halves, respectively, with a 10 mm gap from the external surface of the autoclave (shown in Figure 1b). The height of high-temperature heater (HT heater) is 155 mm, and the height of low-temperature heater (LT heater) is 177 mm. The gap between them is 25.5 mm. The autoclave is made of a nickel base alloy whose trademark is Rene 41. O represents the original point and is located at the cross point of symmetry and the external surface of the autoclave. The axis direction is shown in the figure. Four points’ coordinates and two lines are marked in the figure and will be used to calculate the average external temperature in the discussion part. The materials of the rest of the components and geometric sizes are listed in Figure 1.

### 2.2. Governing Equations

Multi-physics fields and their couplings are calculated in the numerical simulation, including flow, heat transfer, and diluted mass transport. The corresponding differential equations can be acquired from [24]. Bossinesq approximation is employed for the calculation of natural convection. Dissolution and crystallization reactions occur in the nutrient basket and on the surface of seed crystals, respectively. Expressions (1) and (2) express the chemical reactions. According to experimental research, the maximum solubility of metastable GaN with a mineralizer of KNH_2_ in ammonia is determined by temperature [25]. The maximum mass concentration is calculated by Equation (3). The mass source of the diluted mass transport equation includes the dissolution and crystallization reactions and is expressed with Equation (4). It can be inferred that the dissolution and crystallization rates are determined by the chemical kinetics and supersaturation.

(KNH_2_ + NH_3_)_solvent_ + GaN_solid_ → KGa(NH_2_)_4metastable_ (1)

KGa(NH_2_)_4metastable_ → (KNH_2_ + NH_3_)_solvent_ + GaN_solid_ (2)

(3) $$C_{\max}\;=\;\rho_{f}11.7\exp(-0.0122T)/M_{\mathrm{meta}}$$

(4) $$\phi_{c}\;=\;\frac{1}{\forall }[\rho_{f}\kappa A_{\mathrm{fs}}(C_{\max}\;-\;C_{f})]$$

where A_fs_ (SI: m^2^) is the surface and ∀ (SI: m^3^) is the volume. κ represents the chemical reaction coefficient, and the value of 10^−6^ m/s is used here. C_max_ (SI: mol/m^3^) is the maximum mass concentration of metastable GaN. ρ_f_ is the fluid density. C_f_ (SI: mol/m^3^) is the mass concentration of metastable GaN. M_meta_ (SI: kg/mol) is the molecular weight of metastable GaN. *ϕ*_c_ (SI: mol/m^3^) is the mass source. Thus, the supersaturation of metastable GaN can be calculated by C_f_ − C_max_.

All of the physical properties used in the simulation were acquired from [18].

### 2.3. Numerical Setup and Boundary Conditions

The simulation work was finished with the commercial software COMSOL Multiphysics. Modules used in the calculation include non-isothermal flow, Brinkman equation, and diluted mass transport in porous media. The Rayleigh number of the model was calculated using Equation (5). R/2 was adopted as the characteristic length. The value of the Rayleigh number is 5.0 × 10^7^, which is smaller than the critical value. Thus, laminar flow is set in the flow field calculation. Boussinesq approximation was employed as the calculation method of the flow field. Thus, volume force needed to be calculated. The volume force term was set using Equation (6). The whole model is meshed with triangle grids. The total numbers of elements of the basic and optimized model were about 27,571 and 28,733. Five boundary layer grids were set near the internal surface, middle baffle, and seed crystal surface. The time step of the quick calculation was 0.005 s. The second-order backward difference method was applied to discretize the momentum, energy, and mass transport equations. A tolerance error of 0.01 was applied for all the calculated variables at each time step. The liquid ammonia was considered incompressible, and the pressure, viscosity, and thermal capacity were set constant due to their little variation during the growth process. The cost of the computer and time for the calculation were 8 PC cores and 200 h. To confirm that the multi-physics fields in the autoclave reached a relatively steady state, the growth time of 10 h was calculated by a transient calculation.

(5) $$\mathrm{Ra}\;=\;\frac{(T_{H}\;-\;T_{L}){\left(R/2\right)}^{3}}{\upsilon \kappa }$$

F = ρ_ref_ g(T − T_f_) (6)

Here, Ra represents the Rayleigh number. T_H_ is the crystallization temperature, and T_L_ is the dissolution temperature. R is the internal radius of the autoclave (SI: m). υ is the dynamic viscosity (SI: N · s/m^2^). κ is the thermal expansion coefficient of ammonia (SI: 1/K).

Resistance heaters were employed to supply heat sources for the updated ammonothermal system. The heater powers were ascertained by the BP neural net and NSGAII algorithm, presented in Section 3. The bottom of the autoclave was insulated, and a convection boundary with air was employed for the rest of the surface. The internal wall and surface of the baffle in the chamber were set as no-slip walls. The mass source was calculated according to the temperature and mass concentration (Equations (3) and (4)). There was no mass flux for all the internal walls in the chamber (Equation (7)) except the surface of the seed.

(7) ∇c = 0

### 2.4. BP Neural Net and NSGAII Algorithm

In this study, a BP neural network is used first to identify the relationship between heater powers and temperatures of growth and dissolution regions. Then the non-dominated sorting genetic algorithm (NSGAII) was adopted to find the optimal power values for crystal growth. The commercial software MATLAB was employed to realize both of the algorithms.

**Figure 2:**
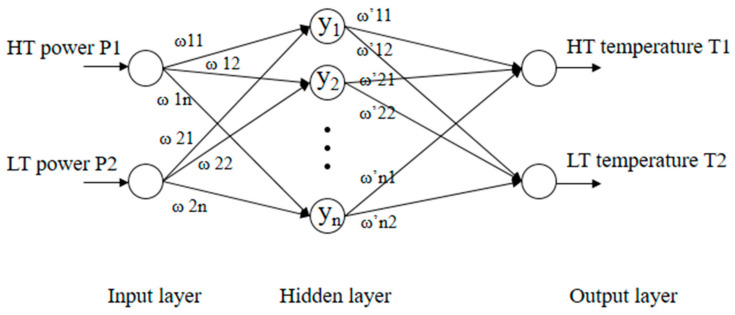
Structure of the three-layer BP network.

To find the optimal power values of heaters, the functional relationship was needed. However, as is known, the ammonothermal autoclave is a complex system, including multi-physics fields and their coupling with each other. There are many factors influencing the temperatures in the dissolution and crystallization region, such as heater powers, boundary conditions, component location in the chamber, and chamber size. Thus, it is not easy to identify the relationship between the powers and temperatures with a simple expression. However, the BP neural network is suitable for such a case. The structure of a three-layer BP network is shown in Figure 2. P_1_ and P_2_ (powers of the high- and low-temperature heaters) are the two input layer nodes. *ω_ij_* represents the weight values between the input layer and hidden layer, *i* = 1, 2; *j* = 1, 2, …, *n*. T1 and T2 (the corresponding temperatures of dissolution and crystallization regions) are the two output layer nodes. *ω_jk_* represents the weight values between the input layer and hidden layer, *j* = 1, 2, …, *n*; *k* = 1, 2. In Figure 2, the HT power P_1_ and LT power P_2_ represent the powers of high- and low-temperature heaters. HT temperature T1 and LT temperature T2 represent the average external surface temperatures in the growth and dissolution regions.

The BP neural net process mainly contains two parts: the signal’s propagation and the output error’s backpropagation [26]. The first part is in the forward direction, which is from the input to the output. The process can be expressed by Equation (8). The second part is in the backward direction, which is from the output to the input. The function of this step is to amend the weight or threshold values to force the results to approach the target values. The error calculation and correction of weight and threshold are expressed by Equations (9)–(13):

(8) $$T_{k}\;=\;f(\sum_{j=1}^{n}\omega_{\mathrm{jk}}\phi (\sum_{i=1}^{2}\omega_{\mathrm{ij}}P_{i}\;+\;\theta_{j})\;+\;a_{k}),k\;=\;1,2$$

(9) $$E\;=\;\frac{1}{2}\sum_{p=1}^{P}\sum_{k=1}^{2}{\left({T_{k}^{p}}^{\prime }\;-\;T_{k}^{p}\right)}^{2}$$

(10) $${\Delta \omega }_{\mathrm{ki}}\;=\;\eta \sum_{p=1}^{P}\sum_{k=1}^{2}\left({T_{k}^{p}}^{\prime }\;-\;T_{k}^{p}\right)\;\cdot \;\psi^{\prime }{(\mathrm{net}}_{k})\;\cdot \;y_{i}$$

(11) $${\Delta a}_{k}\;=\;\eta \sum_{p=1}^{P}\sum_{k=1}^{2}\left({T_{k}^{p}}^{\prime }{\;-\;T}_{k}^{p}\right)\;\cdot \;{\psi^{\prime }(\mathrm{net}}_{k})$$

(12) $${\Delta \omega }_{\mathrm{ij}}\;=\;\eta \sum_{p=1}^{P}\sum_{k=1}^{2}\left({T_{k}^{p}}^{\prime }{\;-\;T}_{k}^{p}\right)\;\cdot \;{\psi^{\prime }(\mathrm{net}}_{k})\;\cdot \;\omega_{\mathrm{jk}}\cdot \phi^{\prime }{(\mathrm{net}}_{j})\;\cdot \;P_{i}$$

(13) $${\Delta \theta }_{j}\;=\;\eta \sum_{p=1}^{P}\sum_{k=1}^{2}\left({T_{k}^{p}}^{\prime }{\;-\;T}_{k}^{p}\right)\cdot {\psi^{\prime }(\mathrm{net}}_{k})\;\cdot \;\omega_{\mathrm{jk}}\;\cdot \;\phi^{\prime }({\mathrm{net}}_{j})$$

where θ_j_ and a*_k_* represent the threshold value of each layer. P is the number of the training samples. η is an adjustment coefficient used to improve the accuracy of weight and threshold values. ψ′ and *ϕ*′ are the functional relationships of hidden layers. net_k_ and net_j_ are the middle values in the neural network.

**Figure 3:**
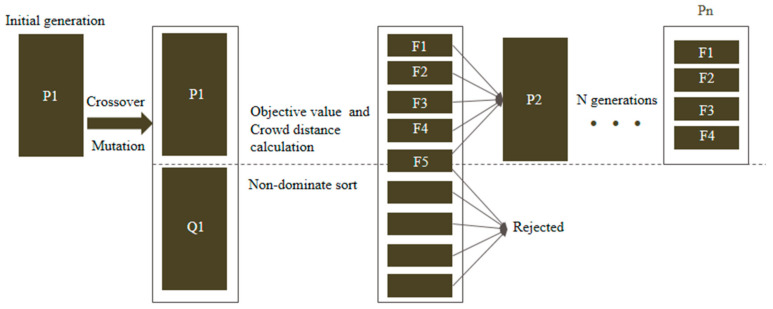
Schematic diagram of NSGAII.

In order to find the optimal heater powers of the ammonothermal system, a non-dominated sorting genetic algorithm (NSGAII) was employed. The principle of the algorithm is shown in Figure 3. An initial population P1 and parent population Q1 were first created by the random method. Then, an equal-sized population was created by crossover and mutation. The two populations were merged, and the non-dominated sorting was conducted by the objective function and crowd distance calculation. Candidates with smaller front values remained, and those with bigger front values were rejected for the next iteration. F*_i_* represents the stochastic frontier in which candidates cannot dominate each other. A total of N generation calculations were operated until the accuracy of the candidates meets the requirements [27,28].

All the candidates were sorted into several frontiers according to the non-dominated method (Figure 3). The frontiers at the front were selected for the next generation until the number of next generation reached N. When the next population could not accommodate all the candidates in F*_i_*, only part of them could be selected. To keep their uniform distribution, they were selected by crowd distance (Equation (15)). Such steps were continued until only one frontier was left.

The objective functions are defined by the deviation of predicted values and objective values (shown as Equations (14) and (15)). Crowd distance is used for candidate selection when they are in the same stochastic frontier. Its expression is shown in Equation (16).

(14) $$f_{1}\;=\;\Delta T_{1}\;=\;{\left(T_{1}\;-\;{T_{1}}^{\prime }\right)}^{2}$$

(15) $$f_{2}\;=\;\Delta T_{2}\;=\;{\left(T_{2}\;-\;{T_{2}}^{\prime }\right)}^{2}$$

(16) $$C_{d}^{k}\;=\;\frac{f_{1}^{k+1}{\;-\;f}_{1}^{k\;-\;1}}{f_{1}^{\max}{\;-\;f}_{1}^{\min}}\;+\;\frac{f_{2}^{k+1}{\;-\;f}_{2}^{k\;-\;1}}{f_{2}^{\max}{\;-\;f}_{2}^{\min}},k\;=\;1,2,\ldots n.$$

Here, is the crowd distance of candidate k. f^max^ and f^min^ are the maximum and minimum objective function values in a particular stochastic frontier. *n* is the number of candidates in the stochastic frontier. T_1_′ and T_2_′ are the target values of dissolution and crystallization temperatures.

## 3. Results and Discussion

### 3.1. Searching for the Optimal Heater Powers

To train the neural network, datasets of heater powers and corresponding temperatures were collected by numerical simulation. The corresponding relationship between heater powers and temperatures is shown in Table 1 and Table 2. The high-temperature heater power values ranged from 450 W to 490 W, and the low-temperature heater power values ranged from 300 W to 390 W. The stable external surface temperature in the crystallization region at certain high- and low-temperature heater power values ranged from 773.5 K to 829.8 K. The stable external surface temperature in the dissolution region at such values ranged from 732.3 K to 799 K.

**Figure 4:**
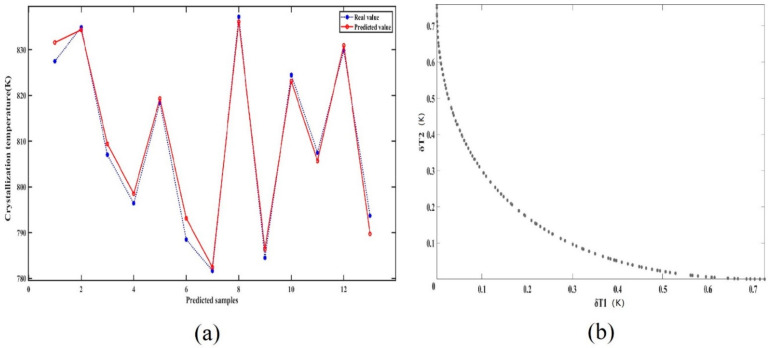
(**a**) Comparison of test values and predicted values. (**b**) Distribution of target function values for the last generation.

Data in the tables above were used to train the neural net. There were nine neural cells in the neural net. The number of training generations was 1000. The target error of the model was set as 0.001. Thirteen sets of data were randomly chosen to test the model. A comparison of real and predicted values is depicted in Figure 4a. The determination coefficient (R^2^) was above 98%, indicating high model accuracy. Such accuracy satisfies the requirement of simulation and experiment.

According to the ammonothermal system used in this study, the optimal dissolution and crystallization temperatures were 798 K and 748 K, respectively. Therefore, they were set as the target temperatures. There were 101 candidates in each generation, and a total of 1000 generations were iteratively computed and non-dominantly sorted by target function values and crowd distance. The distribution of two target function values is shown in Figure 4b. It can be concluded that both temperature differences can be maintained within 0.5 K. It is impossible to find a power set that makes the dissolution and crystallization temperature errors the least at the same time. To reduce the total error, the power set that results in similar values of target errors was selected. According to the last generation, the powers of high- and low-temperature heaters were 300 W and 492 W, respectively.

### 3.2. Heat and Mass Transfer Comparison of Original and Updated Models

**Figure 5:**
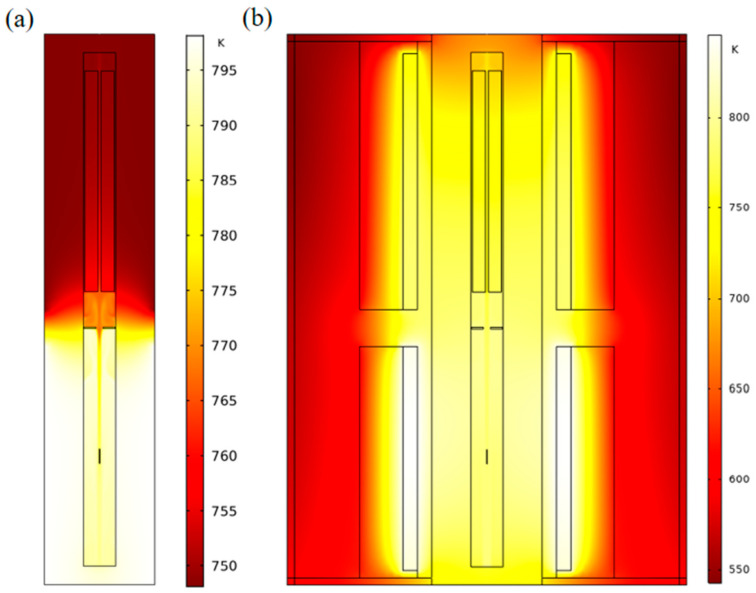
Thermal maps of ammonothermal systems. (**a**) The original simplified system. (**b**) The updated system with resistance heaters.

**Figure 6:**
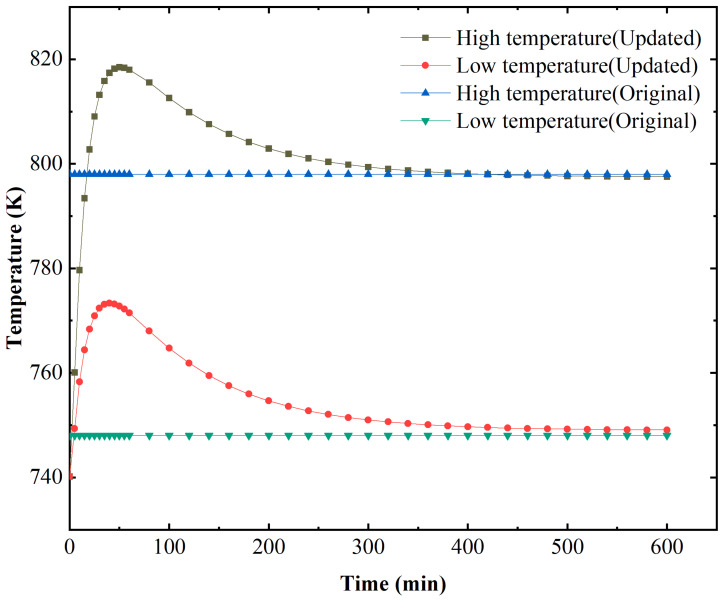
The average temperature variation versus growth time on the external surface of the autoclave.

The heat and mass transfer processes significantly influence the GaN crystal growth. The dissolution and crystallization reaction rates are mainly determined by temperature, and the solute concentration gradient near the crystal surface is maintained by flow and mass transfer in the chamber. There are apparent differences in flow, thermal, and concentration fields between the simplified and updated models. Figure 5 depicts the thermal maps of simplified and updated systems. The temperature boundaries were constant for the simplified system, and the temperature distribution in the dissolution and crystallization regions was more uniform. Meanwhile, for the updated system, the temperatures of the two heaters, as well as the temperature in the chamber, were non-uniform, especially in the top and bottom regions, which agrees with the conclusion of [23]. The temperature distribution has a relatively distinct dividing line in the middle for the simplified system. In contrast, the temperature transition of the updated system was smoother from the lower half to the upper half. Figure 6 shows the average temperature curve on the external surface of the autoclave versus growth time. The X and Y coordinates of the surfaces are from (39, 10) to (39, 165) and from (39, 190.5) to (39, 367.5). For the original point and axis direction, please refer to Figure 1. There is a noticeable fluctuation in temperature for the system with resistance heaters. This is probably because of the imbalance of heat transfer at the beginning of the growth period.

**Figure 7:**
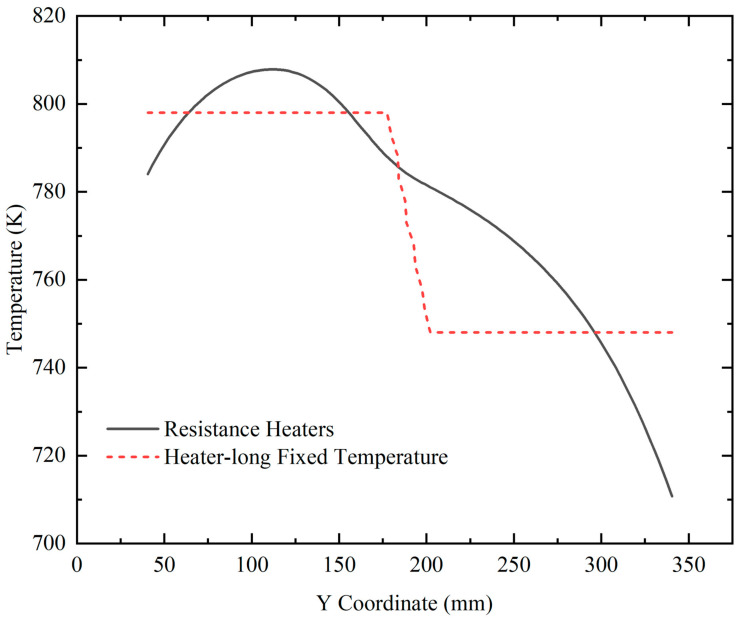
The temperature distribution on the external surface of the autoclave lateral wall versus vertical position.

The natural convection and mass transfer were determined by the temperature difference between the lower and upper halves. Therefore, to verify our simulation result, the stable temperature distribution on the external surface of the autoclave lateral wall where the X coordinate was 39 mm was calculated and compared with the result in [23] (shown as Figure 7). The variation trend of the temperature profile with resistance heaters is almost the same as that in Figure 10 in [23], which was partly verified by temperature measurement on the external surface of the autoclave. However, there was a slight difference at the bottom of the autoclave wall. This is mostly because of the difference in the high-temperature heater location. In [23], the high-temperature heater was located lower than the bottom of the chamber. Therefore, there was no temperature rise along the vertical location at the bottom of the autoclave lateral wall. At the top of the autoclave wall, the temperature declined more in [23], owing to the lack of insulation at the top of the low-temperature heater. The temperature profile with resistance heaters was smooth, and there were significant temperature differences at the bottom and top regions, which agreed well with the conclusion of [23]. As we all know, the real temperature distribution along the external surface of the autoclave should be continuous when it reaches a steady state. Thus, the simulation result with resistance heaters instead of heater-long fixed temperatures is closer to the real situation.

**Figure 8:**
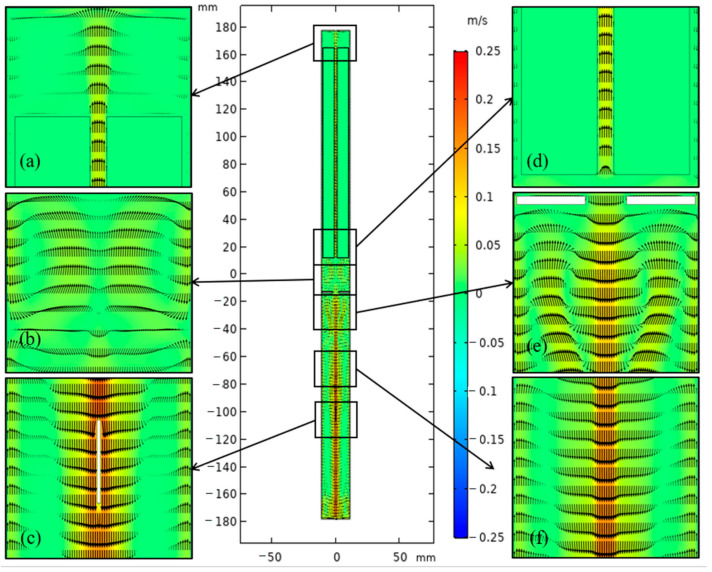
Velocity vector distribution of the ammonothermal system with heater-long fixed temperature boundary. (**a**) Part 1. (**b**) Part 2. (**c**) Part 3. (**d**) Part 4. (**e**) Part 5. (**f**) Part 6.

**Figure 9:**
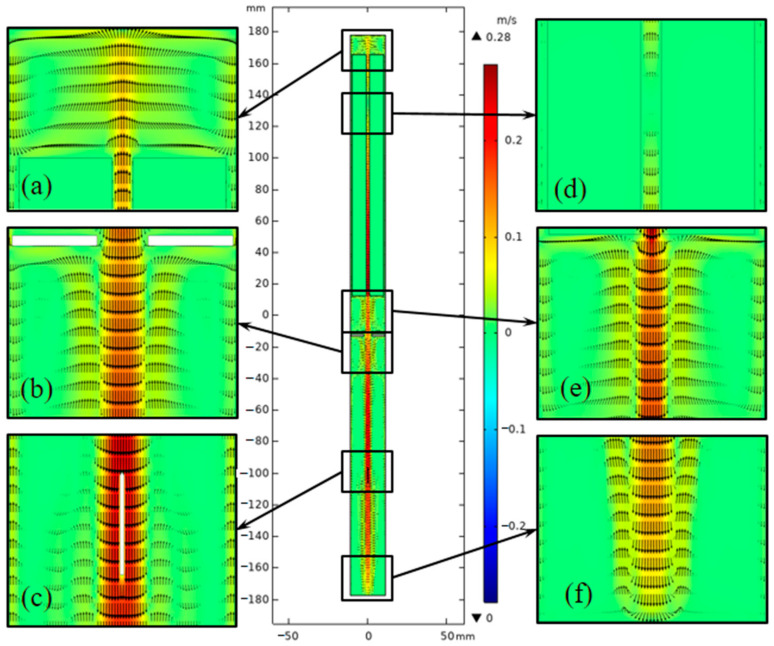
Velocity vector distribution of the ammonothermal system with resistance heaters as the heat source. (**a**) Part 1. (**b**) Part 2. (**c**) Part 3. (**d**) Part 4. (**e**) Part 5. (**f**) Part 6.

The flow fields of original and updated cases are depicted in Figure 8 and Figure 9. To clearly show the details of flow and thermal fields in the chamber, six enlarged drawings (Part 1–Part 6) were added. The flow style in the lower half of the chamber was similar to cases A3 and B in [23]. Apparent discrepancies in the velocity vector distribution between the two systems were observed, especially at the top and bottom. The temperature gradient in the upper half improved due to the convection heat loss at the top of the system. Thus, flow above the nutrient basket was stronger for the updated system than for the original system. However, the flow in the lower half was weakened, as the temperature boundary between the lower and upper half was blurrier than that in the original system. Flow almost disappeared at the bottom of the chamber, which is unfavorable for mass transport. 

**Figure 10:**
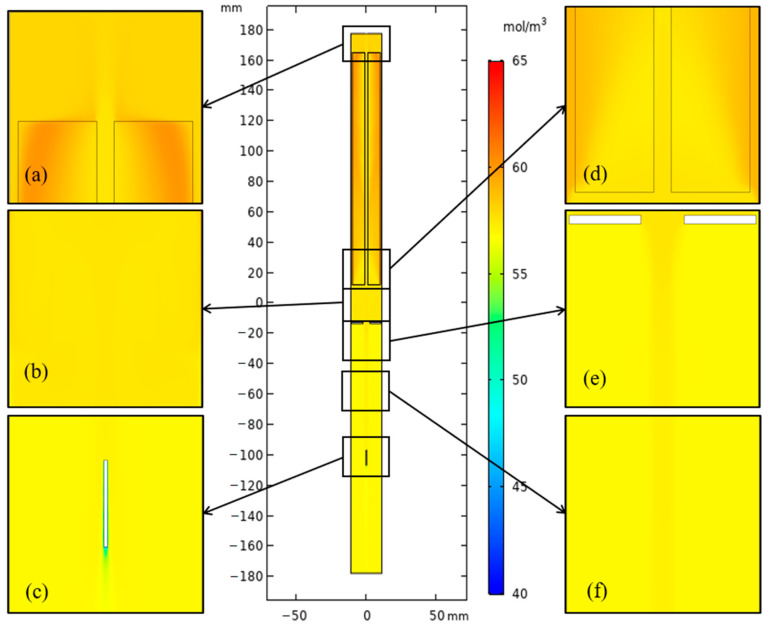
Concentration distribution of the ammonothermal system with heater-long fixed temperature boundary. (**a**) Part 1. (**b**) Part 2. (**c**) Part 3. (**d**) Part 4. (**e**) Part 5. (**f**) Part 6.

**Figure 11:**
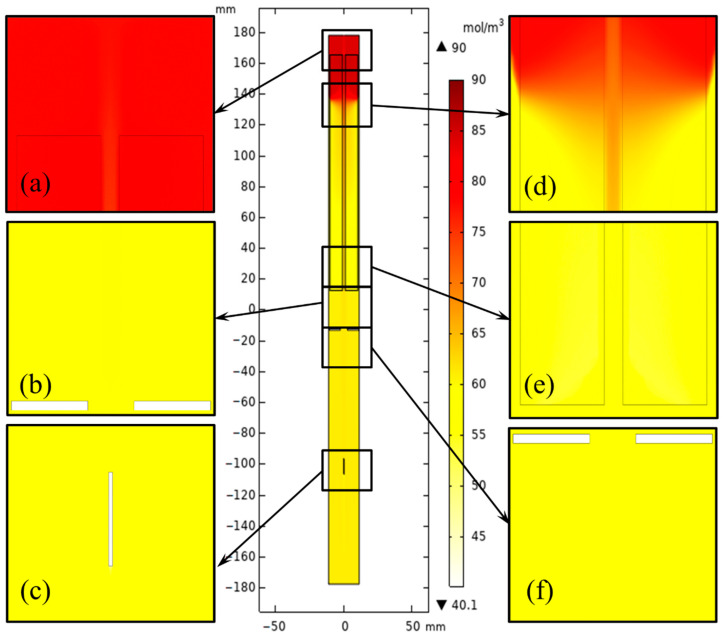
Concentration distribution of the ammonothermal system with resistance heaters as the heat source. (**a**) Part 1. (**b**) Part 2. (**c**) Part 3. (**d**) Part 4. (**e**) Part 5. (**f**) Part 6.

The hot and cool fluid exchange happened mainly in the region between the middle baffle and the nutrient basket for the system with a heater-long fixed temperature boundary. The concentration distribution in the upper half of the chamber varied smoothly at different elevations (shown in Figure 10). However, the velocity magnitude at the region between the middle baffle and nutrient basket was reduced when resistance heaters were employed as heat sources. A high-concentration region at the top of the chamber (Figure 11) appeared as the temperature was reduced by heat loss at the head of the autoclave, which is beneficial for the dissolution of GaN with retrograde solubility in supercritical ammonia. The concentration level of the metastable GaN in the free flow is higher than that in the original model. The heat and mass transfer comparison of the two ammonothermal system models suggests that it is necessary to construct an ammonothermal system model with resistance heaters.

### 3.3. Large-Sized GaN Crystal Growth Analysis

To research the heat and mass transfer in the chamber for large-sized crystal growth, the 2- and 4-inch ammonothermal system models were designed and calculated in proportion, respectively. The powers of heaters were calculated using the method above. The values of power were 1210 W and 1040 W for the 2-inch autoclave and 2430 W and 2255 W for the 4-inch autoclave.

**Figure 12:**
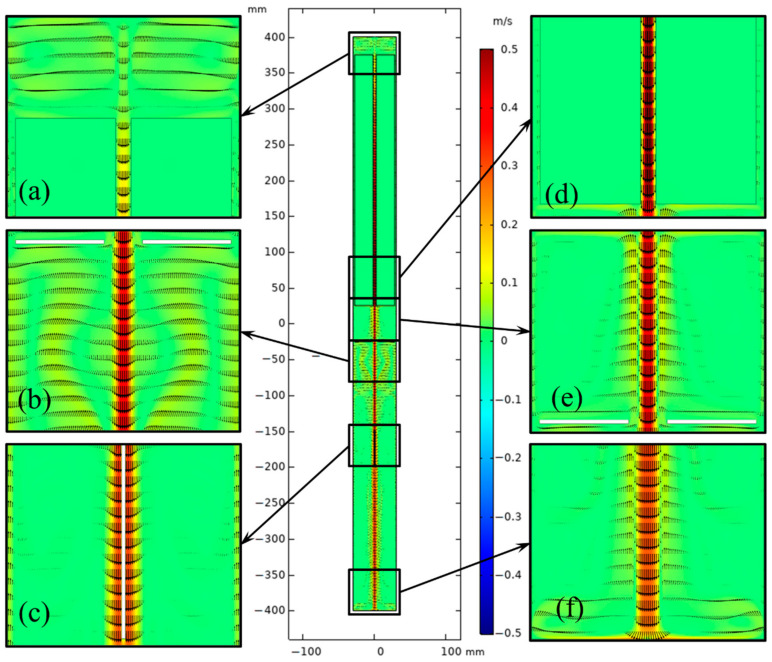
Velocity distribution in the chamber for the 2-inch ammonothermal system. (**a**) Part 1. (**b**) Part 2. (**c**) Part 3. (**d**) Part 4. (**e**) Part 5. (**f**) Part 6.

**Figure 13:**
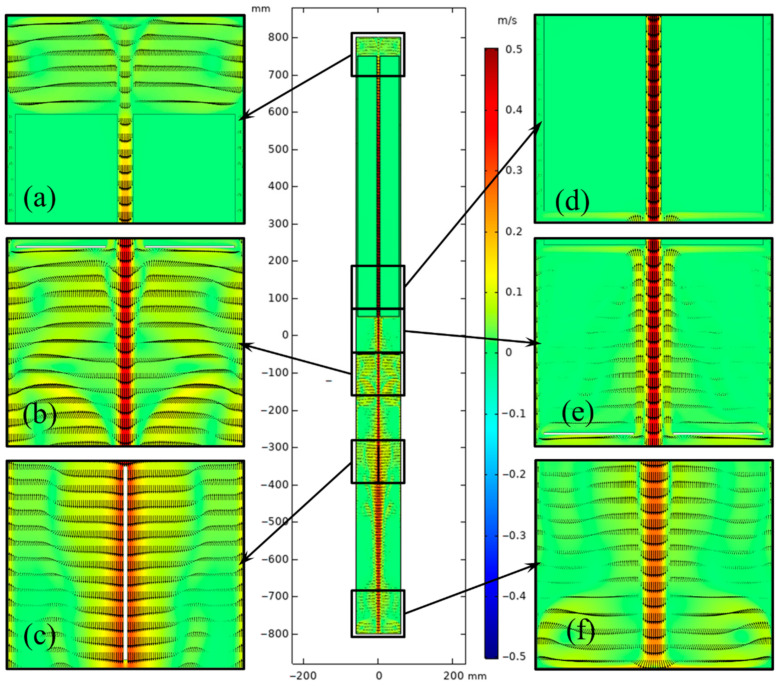
Velocity distribution in the chamber for the 4-inch ammonothermal system. (**a**) Part 1. (**b**) Part 2. (**c**) Part 3. (**d**) Part 4. (**e**) Part 5. (**f**) Part 6.

Figure 12 and Figure 13 depict the flow fields in the chamber for the 2-inch and 4-inch ammonothermal systems. The natural convection was strengthened with increasing size. The velocity of central flow for a large-sized ammonothermal system was higher, which is beneficial for mass transport. With the help of a stronger central flow, convection at the bottom of the chamber was more fierce than that in the smaller-sized system (Figure 9).

**Figure 14:**
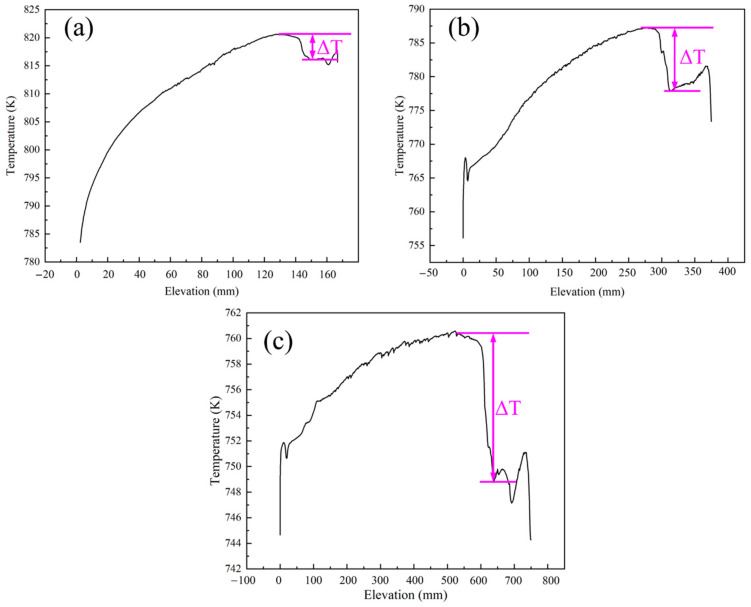
Temperature profile near the internal surface of the autoclave at the lower half of the chamber. (**a**) Curve for the 1-inch system; (**b**) 2-inch system; (**c**) 4-inch system.

According to research by Hooman Enayati et al. [13,14,15,16], the fundamental driver of natural convection in the chamber is the buoyancy at the boundary layer, which is derived from the temperature gradient on the internal surface of the autoclave near the baffle. As there is a nutrient basket in the upper half of the chamber, the temperature gradient in the lower half offers the main force. Figure 14 shows the temperature profile near the internal surface in the lower half. There is a distinct temperature re-duction near the middle baffle. The temperature (ΔT) gradient around the baffle rose as the diameter of the chamber increased. This probably resulted from the increasing inhomogeneity of heat transfer for a larger sized system.

**Figure 15:**
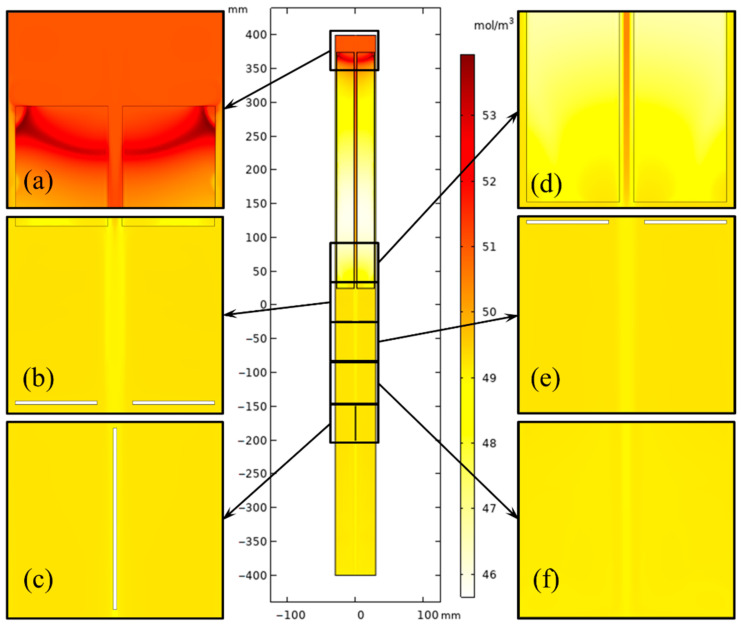
Metastable GaN concentration distribution in the chamber for the 2-inch ammonothermal system. (**a**) Part 1. (**b**) Part 2. (**c**) Part 3. (**d**) Part 4. (**e**) Part 5. (**f**) Part 6.

**Figure 16:**
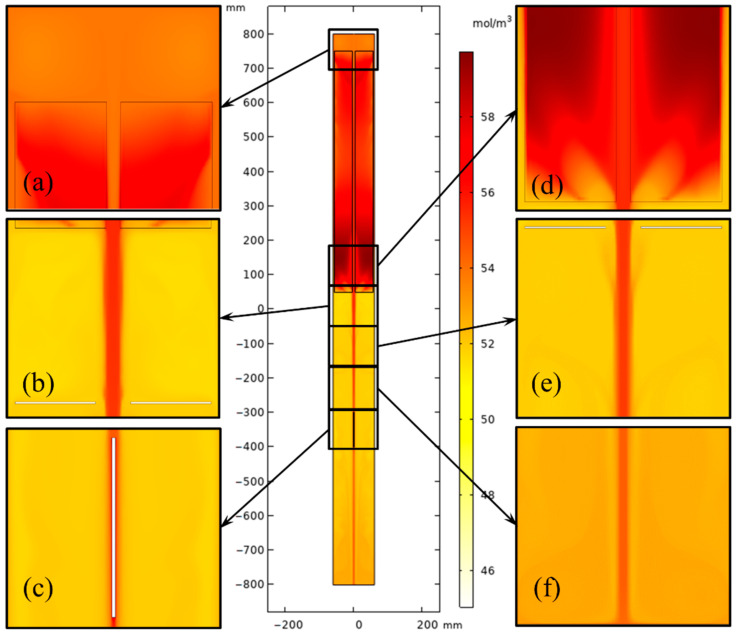
Metastable GaN concentration distribution in the chamber for the 4-inch ammonothermal system. (**a**) Part 1. (**b**) Part 2. (**c**) Part 3. (**d**) Part 4. (**e**) Part 5. (**f**) Part 6.

To analyze the growth rate of GaN in a large-sized ammonothermal system, the concentration distribution and gradient on the seed crystal surface need to be calculated. Figure 15 and Figure 16 show the concentration distribution in the chamber for 2-inch and 4-inch systems. The inhomogeneity of the mass concentration in the nutrient basket was more evident as the internal diameter of the autoclave increased because of the uneven temperature distribution. However, with the help of stronger central flow, the metastable GaN concentration in the growth region (lower half) was improved.

**Figure 17:**
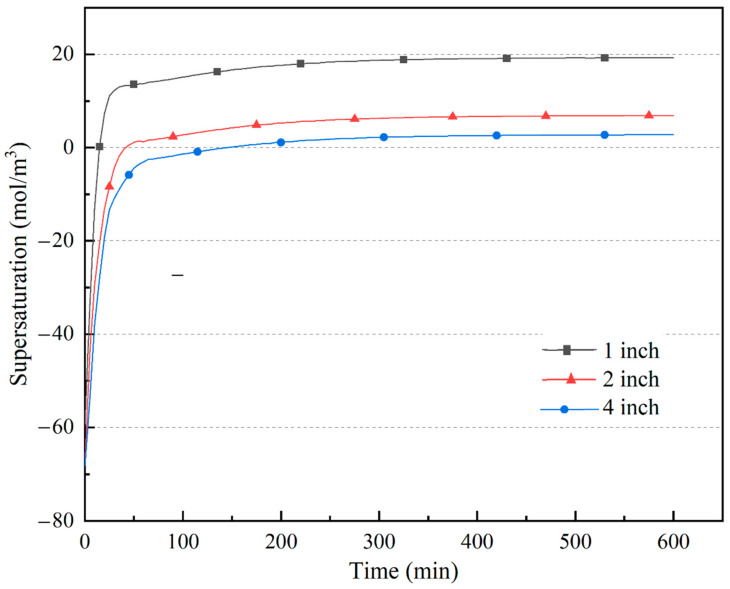
Average metastable GaN supersaturation on the seed crystal surface variation versus growth time.

The primary determining factor of growth rate is the supersaturation on the seed crystal surface at a particular chemical kinetics coefficient. Thus, the variation in supersaturation on the seed crystal surface needs more attention, and it can be calculated by transient simulation. Figure 17 depicts the supersaturation variation versus growth time for the three ammonothermal systems. The increasing rate and maximum value of the average supersaturation on the seed crystal surface were reduced with increasing system size, owing to the temperature decline in the growth region due to stronger natural convection. This resulted in a lower crystal growth rate in a large-sized ammonothermal system.

## 4. Conclusions

The ammonothermal system model was better developed by replacing the heater-long fixed temperature boundary with resistance heaters and considering the real thermal boundary conditions. A BP neural network was used to express the relationship between heater powers and temperatures of dissolution and crystallization. The optimal power values were found by a non-dominated sorting genetic algorithm (NSGAII), which is also applicable for autoclave design and a quick search of heater powers when conducting the experiment.

The simulation result was compared with that in [23] for verification. The conclusion in this paper agrees well with that in [23], which was partly verified by measuring the temperature at several points. With a better model, it is possible for us to better understand the heat and mass transfer in the ammothermal system, which can not be measured and observed directly for now. This paper supplied a new method for the lower-cost and less time-consuming optimization of the ammonothermal system, and the method can also be used to control the growth parameters in commercial production in the future.

A comparison of the original and updated simulations shows several differences between heat and mass transfer. The flow of the well-developed system was stronger at the top and weaker at the bottom. A higher concentration region appeared in the upper part of the nutrient basket with the influence of heat loss at the head of the autoclave. The metastable GaN concentration in the crystallization region was higher than that in the original model. Therefore, it is necessary to conduct the simulation work with an updated ammonothermal system model.

Based on the better-developed ammonothermal system, large-sized crystal growth was analyzed. Models of 2-inch and 4-inch ammonothermal systems were built. Calculation results show that the natural convection was strengthened as the size increased because of the higher inhomogeneity of heat transfer. With the influence of stronger central flow in the crystallization region, the metastable GaN concentration in the lower half of the chamber was improved. However, the increasing rate and maximum stable value of average supersaturation on the seed crystal surface were reduced owing to the temperature decline caused by the central flow, which results in a lower growth rate for a larger-sized ammonothermal system. Thus, it needs optimization for the large-sized system to improve the homogeneity of heat transfer in the autoclave for a better growth environment.

## Figures and Tables

**Table 1:** Crystallization temperature (unit: K) of crystallization region at different power values.

	High-Temperature Heater Power (W)					
Low-temperature heater power (W)		450	460	470	480	490
	300	773.5	778.9	784.7	790.2	796.4
	310	776.9	781.66	788.5	794.3	800
	330	784.5	790.3	796	801.7	807.5
	350	792	797.84	803.6	809.3	814.9
	370	799.5	805.3	811	816.7	822.4
	390	807	812.7	818.4	824.5	829.8

**Table 2:** Dissolution temperature (unit: K) of crystallization region at different power values.

	High-Temperature Heater Power (W)					
Low-temperature heater power (W)		450	460	470	480	490
	300	732.3	736.3	740.3	744.5	748.3
	310	738.1	742.1	746	750	754
	330	749.7	753.6	757.5	761.5	765.4
	350	761.2	765.1	769	772.8	776.66
	370	772.5	776.4	780.2	784	787.9
	390	783.8	787.6	791.45	795.5	799

## Nomenclature

ρ_f_	fluid density (kg/m^3^)
C_max_	maximum concentration (mol/m^3^)
T	temperature (K)
M_meta_	molecular weight of the metastable species (kg/mol)
$\phi_{c}$	mass source (mol/(m^3^s))
$A_{\mathrm{fs}}/\forall$	surface-to-volume fraction (1/m)
κ	reaction rate coefficient (m/s)
c & Cf	species concentration (mol/m^3^)
T_H_	crystallization temperature (K)
T_L_	dissolution temperature (K)
R	internal radius of the autoclave (m)
υ	dynamic viscosity (N · s/m^2^)
κ	thermal expansion coefficient (1/K)
Ra	Rayleigh number
ω	weight values
a & θ	threshold values
f1 & f2	objective function
Cd	crowd distance
